# Cardiac mechanical activation mapping in heart failure patients with left bundle branch block using cine DENSE MRI

**DOI:** 10.1186/1532-429X-17-S1-O43

**Published:** 2015-02-03

**Authors:** Daniel A Auger Cornejo, Sophia Cui, Xiao Chen, Kenneth C Bilchick, Frederick H Epstein

**Affiliations:** 1 Department of Biomedical Engineering, University of Virginia, Charlottesville, VA, USA; 2 Department of Medicine, Cardiovascular Medicine, University of Virginia, Charlottesville, VA, USA

## Background

Cardiac resynchronization therapy (CRT) is an effective treatment for selected patients with heart failure (HF) and left bundle branch block (LBBB). However, an ongoing major issue with CRT is that 30-50% of treated patients are non-responders. One potential cause of a poor response is implantation of the CRT left-ventricular (LV) pacing lead at a suboptimal location, i.e., a location with scar or where mechanical activation is not delayed [1,2]. This study developed and applied cine DENSE strain imaging [3] to map mechanical activation and detect late-activated segments.

## Methods

**Figure 1:**
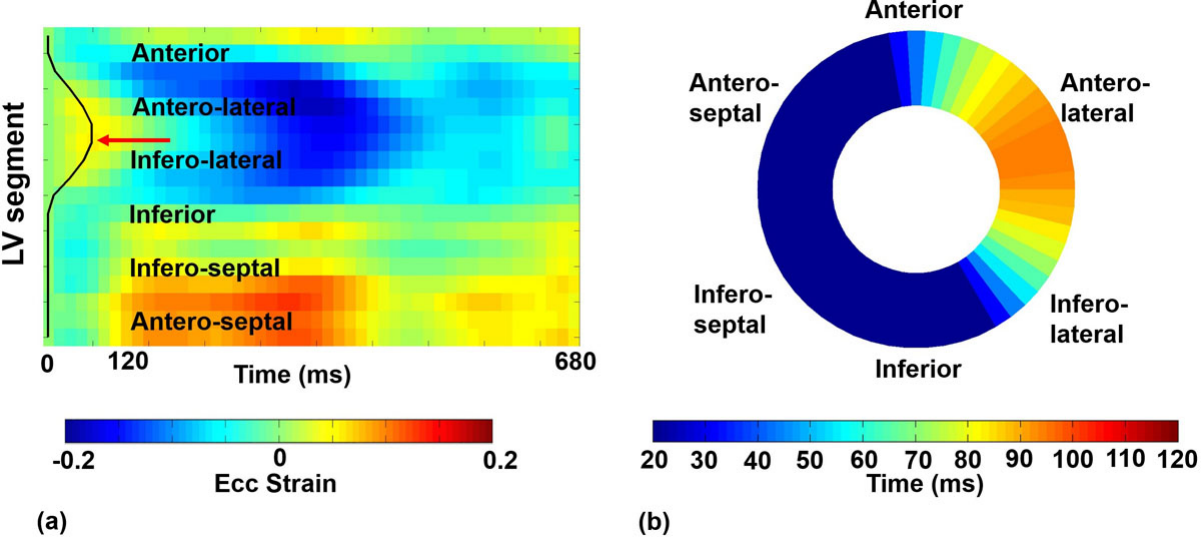
(a) A strain matrix reveals pre-stretch and late activation of the lateral wall (red arrow) in a patient with heart failure and LBBB. An active contour (black line) detects the mechanical activation times for all LV segments. (b) The corresponding activation time map shows late activation of the anterolateral wall.

Cine DENSE was performed on a 1.5T MRI system in standard short-axis planes in 6 healthy subjects and 16 HF patients with LBBB and nonischemic cardiomyopathy referred to CRT. Circumferential strain (Ecc) was computed using previously described semiautomatic methods [4,5]. Midwall Ecc was arranged into a matrix with 18 rows representing spatial segments of the LV and 30-45 columns representing cardiac phases (Fig 1a). Singular value decomposition (SVD) was applied to denoise the spatiotemporal Ecc matrix, and an active contour method was used to automatically estimate the time to the onset of shortening, which was defined as the mechanical activation time. During the CRT implementation procedure, the electrical activation time (QLV) was assessed at the LV lead implantation site.

## Results

**Figure 2:**
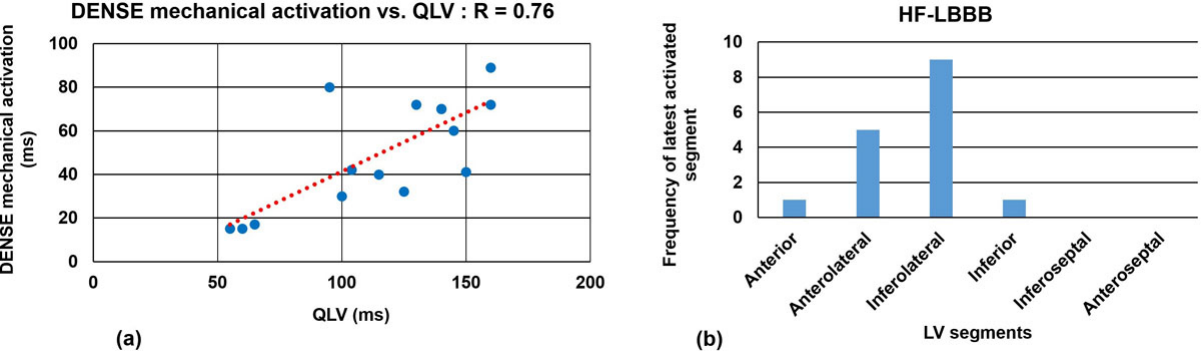
(a) Correlation of DENSE mechanical activation time and electrical activation time measured by qLV. (b) The distribution of the location of the latest-activating segments.

Fig 1(a) shows an Ecc matrix of a patient with HF and LBBB. A region of pre-stretch and delayed activation is evident in the lateral wall (red arrow), and the delayed activation time is accurately detected by the active contour (black line). The corresponding activation time map is shown in Fig 1(b), where activation of the lateral wall is delayed by 90 ms. The mean time of latest mechanical activation was 70 ± 19.8 ms in HF patients compared to 26 ± 7 ms in healthy subjects (p<0.01). Fig 2(a) shows the correlation between QLV and DENSE mechanical activation time at matched locations. Variations in the region of the latest mechanical activation among patients are shown in Fig 2(b).

## Conclusions

Cine DENSE strain imaging detects late-activated segments in HF patients with LBBB referred to CRT. Mechanical activation delays correlate with electrical activation times measured at matched locations. Heterogeneity in the location of late-activating segments suggests that activation mapping holds potential for pre-procedure identification of optimal LV lead implantation sites for individual patients undergoing CRT.

## Funding

This research was funded in part by NIH R01 EB001763 and Siemens.
